# Macadamized Streets

**Published:** 1881-07

**Authors:** 


### MACADAMIZED STREETS.

The St. Louis *Globe-Democrat* indulges in some strong language on the subject of macadamized streets. No language can be too strong to do justice to the subject, when the streets which are covered with the invention of the Scotchman whose name has come down from a former generation in connection with the road covering, which when of good hard trap or granite makes the best of rural roads, but when of friable stone and cared for after the American fashion, become to the dwellers in St. Louis, Cincinnati, and upper Fifth avenue a source of unspeakable discomfort and expense.

It would be difficult to find a material better calculated to irritate the eyes, the throat, and the lungs, not to speak of the feelings of the unfortunates who have to encounter it, than the fine dust which is ground from a macadamized city street, when not properly cared for. And where in the United States is there a city which takes proper care of its streets? This dust when dry is whirled aloft into the eyes and mouths of passengers, and into every corner of the house. When moistened, it makes a nasty paste, dangerous to horses and inconvenient to pedestrians, and destructive to the clothing on which it is spattered. It may be that when under the regime proposed to be established by the new street cleaning bill, the streets of New York are swabbed and holystoned every morning, a macadamized street will be endurable, but under the administration of the past and the present, whether here or in the West, the less of that kind of pavement that is laid the better will the streets of our cities be.—*Sanitary Engineer.*

